# Pregnancy and childbirth outcomes among indigenous adolescents in Guatemala: a cohort study

**DOI:** 10.1186/s12978-022-01421-x

**Published:** 2022-06-23

**Authors:** Noe Gómez, Odette Del Risco Sánchez, Maira Pinho-Pompeu, Helymar Machado, Luis Bahamondes, Fernanda Surita

**Affiliations:** 1 Department of Obstetrics and Gynecology, State University of Campinas, Av. Alexander Fleming, 101–101 Alexander Fleming Street, Campinas, SP 13083-881 Brazil; 2 Department of Obstetrics and Gynecology, Hospital Regional San Juan De Dios, Quetzaltenango, Guatemala

**Keywords:** Indigenous women, Adolescent pregnancy, Reproductive health, Vulnerability, Guatemala, Mulher indígena, Gestação na adolescência, Saúde reprodutiva, Vulnerabilidade, Guatemala, Mujeres indígenas, Embarazo adolescente, Salud reproductive, Vulnerabilidad, Guatemala

## Abstract

**Objective:**

To assess some characteristics and outcomes associated with pregnancy among Indigenous adolescents and compare them with other women who gave birth in a public hospital in Guatemala.

**Methods:**

We conducted a retrospective cohort study of 8048 cases. Sociocultural variables, gynecological and obstetric history, childbirth, and perinatal outcomes were compared among women who gave birth at San Juan De Dios Hospital between January 2018 and June 2019. They were classified into four groups according to age and ethnicity. Indigenous adolescents (819/10.2%) were compared with Nonindigenous adolescents (813/10.1%), Indigenous adult women (3324/41.3%), and Nonindigenous adult women (3092/38.4%). Bivariate analysis and multiple logistic regression were applied.

**Results:**

We found that Indigenous adolescents who gave birth in the public hospital had fewer years of schooling than Nonindigenous adolescents (p < 0.001), Indigenous adults (p < 0.001), and Nonindigenous adults (p < 0.001). Indigenous adolescents were more likely to have an unplanned pregnancy than Nonindigenous adolescents (p = 0.038) and Nonindigenous adults (p < 0.001) and were more likely to be single (p < 0.001) and use less previous contraception than Indigenous and Nonindigenous adult women (p = 0.007 and p = 0.013, respectively). More than one-third of Indigenous adolescents and adults did not attend antenatal care; Indigenous adolescents had fewer antenatal care visits than Nonindigenous adults (p < 0.001), and the results were borderline in comparison to Nonindigenous adolescents (p = 0.051). Indigenous and Nonindigenous adult women underwent episiotomy less often than Indigenous adolescents (OR: 0.60 [95% CI 0.49–0.74] and OR: 0.56 [95% CI 0.45–0.70], respectively) and received less local anesthesia than Indigenous adolescents (OR: 0.59 [95% CI 0.46–0.76] and OR: 0.77 [95% CI 0.60–0.99], respectively). Nonindigenous adults received more analgesia than Indigenous adolescents (OR: 1.36 [95% CI 1.07–1.73]). Nonindigenous adolescents had more newborns with low birth weight than Indigenous adolescents (OR: 1.44 [95% CI 1.10–1.87]).

**Conclusion:**

Indigenous adolescents who gave birth in a public hospital in Guatemala were more likely to be single during pregnancy and attend fewer years of school than Nonindigenous adolescents. Unplanned pregnancies were more common among Indigenous adolescents, and some of them underwent not recommended obstetric practices during childbirth, such as episiotomy. Police should be enforced ensuring equal opportunities for different ethnic and age groups regarding pregnancy.

## Plain Language Summary

This study aims to evaluate the sociodemographic characteristics and outcomes associated with pregnancy in Indigenous adolescents and compare them to other women who gave birth in a public hospital in Guatemala. We conducted a retrospective cohort study on four groups: Indigenous adolescents, Nonindigenous adolescents, Indigenous adults, and Nonindigenous adults. Subsequently, we compared the sociodemographic characteristics of the latter three groups with those of the Indigenous adolescents to evaluate whether being in this age group in combination with belonging to an Indigenous ethnic group increased unfavorable outcomes during pregnancy, childbirth, and postpartum. We observed that Indigenous adolescents have limited academic opportunities compared with Nonindigenous adolescents; the usage of contraceptive methods was lower in adolescents (Indigenous and non-Indigenous), and unplanned pregnancies were more frequent in Indigenous adolescents than in Nonindigenous adolescents and adults), and a high percentage of all women did not attend antenatal care. We analyzed obstetric practices during childbirth, and a high incidence of episiotomies was reported in both groups of adolescents (42.5% for Indigenous women and 38.8% for Nonindigenous women). Newborns of Nonindigenous adolescents had a higher frequency of low birth weight. Our study provides an overview of the characteristics of pregnancy among different age groups, findings that could be used to develop targeted interventions for each group and create public policies that would provide equal opportunities for all women while also ensuring a healthy pregnancy.

## Resumo

**Objetivo:**

Avaliar as características e os desfechos associados à gravidez em adolescentes indígenas e compará-los com mulheres que tiveram o parto em um hospital público da Guatemala.

**Métodos:**

Estudo de coorte retrospectivo com 8,048 casos. Variáveis socioculturais, histórico ginecológico e obstétrico, dados referentes ao parto e resultados perinatais foram comparados entre as mulheres que deram à luz no Hospital San Juan De Dios entre janeiro de 2018 e junho de 2019. As mulheres foram classificadas em quatro grupos; segundo idade e etnia. As adolescentes indígenas (819/10.2%) foram comparadas com adolescentes não indígenas (813/10.1%), adultas indígenas (3324/41.3%) e adultas não indígenas (3092/38.4%). Análise bivariada e regressão logística múltipla foram realizadas.

**Resultados:**

Adolescentes indígenas que deram à luz no hospital público tinham menos anos de estudo em relação às adolescentes não indígenas (p < 0.001), adultas indígenas (p < 0.001) e adultas não indígenas (p < 0.001). Adolescentes indígenas apresentaram maior probabilidade de ter uma gravidez não planejada em relação às adolescentes não indígenas (p = 0.038) e adultas não indígenas (p < 0.001) e maior probabilidade de serem solteiras (p < 0.001) e não usarem contracepção prévia em relação às adultas indígenas e adultas não indígenas (p = 0.007 e p = 0.013, respectivamente). Mais de um terço das adolescentes e adultas indígenas não compareceram ao pré-natal; Adolescentes indígenas tiveram menos consultas de pré-natal em relação às adultas não indígenas (p < 0.001), e os resultados foram limítrofes em comparação com as adolescentes não indígenas (p = 0.051). Mulheres adultas indígenas e não indígenas foram submetidas a episiotomia com menor frequência em comparação as adolescentes indígenas (OR: 0.60 [IC 95%: 0.49–0.74] e OR: 0.56 [IC 95%: 0.45–0.70], respectivamente) e receberam menos anestesia local do que as adolescentes indígenas (OR: 0.59 [IC 95%: 0.46–0.76] e OR: 0.77 [IC 95%: 0.60–0.99], respectivamente). Adultas não indígenas tiveram maior prevalência no uso de analgesia do que as adolescentes indígenas (OR: 1.36 [IC 95%: 1.07–1.73]). Adolescentes não indígenas tiveram maior prevalência de recém-nascidos com baixo peso do que adolescentes indígenas (OR: 1.44 [IC 95%: 1.10–1.87]).

**Conclusão:**

Adolescentes indígenas que deram à luz em um hospital público na Guatemala tiveram maior probabilidade de serem solteiras durante a gravidez e terem menos anos de escolaridade do que as adolescentes não indígenas. Além disso, adolescentes indígenas tiveram mais gestações não planejadas e experimentaram mais práticas obstétricas não recomendadas durante o parto, como a episiotomia. Fiscalizações devem ser aplicadas a fim de garantir oportunidades iguais para diferentes grupos étnicos e etários em relação aos direitos reprodutivos e ao monitoramento da gravidez.

## Resumen

**Objetivo:**

Evaluar algunas características y resultados asociados al embarazo en adolescentes indígenas, y compararlos con otras mujeres que tuvieron partos en un hospital público de Guatemala.

**Métodos:**

Realizamos un estudio de cohorte retrospectivo de 8.048 casos. Se compararon variables socioculturales, antecedentes ginecológicos y obstétricos, así como resultados perinatales y del parto entre mujeres que tuvieron sus partos en el Hospital San Juan de Dios entre enero de 2018 y junio de 2019. Las mujeres fueron clasificadas en cuatro grupos, según su edad y etnia. Se compararon adolescentes indígenas (819/10,2%) con adolescentes no indígenas (813/10,1%), mujeres adultas indígenas (3.324/41,3%) y mujeres adultas no indígenas (3.092/38,4%). Para el análisis estadístico se empleó análisis bivariado y regresión logística múltiple.

**Resultados:**

Las adolescentes indígenas que tuvieron su parto en un hospital público tenían menos años de escolaridad que las adolescentes no indígenas (p < 0,001), las adultas indígenas (p < 0,001) y las adultas no indígenas (p < 0,001). Las adolescentes indígenas tenían más probabilidades de tener un embarazo no planificado que las adolescentes no indígenas (p = 0,038) y las adultas no indígenas (p < 0,001), así como tenían más probabilidades de ser solteras (p < 0,001) y usaban menos métodos anticonceptivos antes del embarazo que las mujeres adultas indígenas y no indígenas (p = 0,007 y p = 0,013, respectivamente). Más de un tercio de las adolescentes y de adultas indígenas no asistieron a consultas prenatales. Las adolescentes indígenas tuvieron menos consultas prenatales que las adultas no indígenas (p < 0,001), y los resultados fueron limítrofes en comparación con las adolescentes no indígenas (p = 0,051). Las mujeres adultas indígenas y no indígenas fueron sometidas a episiotomía con menos frecuencia que las adolescentes indígenas (OR: 0,60 [IC 95%: 0,49–0,74] y OR: 0,56 [IC 95%: 0,45–0,70], respectivamente) y recibieron menos anestesia local que las adolescentes indígenas (OR: 0,59 [IC 95%: 0,46-0,76] y OR: 0,77 [IC 95%: 0,60-0,99], respectivamente). Las adultas no indígenas recibieron más analgesia que las adolescentes indígenas (OR: 1,36 [IC 95%: 1,07–1,73]). Las adolescentes no indígenas tuvieron más recién nacidos con bajo peso al nacer que las adolescentes indígenas (OR: 1,44 [IC 95%: 1,10–1,87]).

**Conclusión:**

Las adolescentes indígenas que tuvieron su parto en un hospital público de Guatemala tenían más probabilidades de estar solteras durante el embarazo y presentar menos años de escolaridad que las adolescentes no indígenas. Los embarazos no planificados fueron más comunes entre las adolescentes indígenas, y algunas de ellas fueron sometidas a prácticas obstétricas no recomendadas durante el parto, como episiotomía. En relación con la asistencia durante el embarazo, se deben implementar medidas que garanticen la igualdad de oportunidades para diferentes grupos étnicos y etarios.

## Introduction

Initiatives to prevent pregnancy among adolescents in Guatemala have yet to achieve their desired impact, as an average of 107,664 adolescents per year reported pregnancies between 2015 and 2019 [1]. The latest estimates report that there were 104,837 adolescent pregnancies in 2020 [2]. Six states reported the highest number of pregnancies; they represented 57.5% of the total cases and had the lowest coverage rates for primary education and healthcare services and the highest poverty rates [2]. In 2014, 23.4% of the Guatemalan population experienced extreme poverty, and 61.6% were considered multidimensionally poor [3]. According to the Multidimensional Poverty Index (MPI), urban communities in Guatemala receive a score of 0.049, and in contrast, this value increases to 0.161 for rural populations, which reveals a large discrepancy between these two areas [4].

In Guatemala, 43.8% of the population are Indigenous [5], having beliefs, practices, and unique languages (one of the 22 Mayan languages in the country) [6], and 25% are in the age group of 10 to 19 years [7]. Most of these Indigenous people are concentrated in the northeastern region of the country in states with a higher total fertility rate. They live in small communities with fewer than 20,000 inhabitants [8], making it difficult to provide education and healthcare services near families. Only 3 out of 10 Indigenous students report that they complete elementary school [9], and the average number of years of schooling for adolescents aged 15 years is 4 years [10].

The main causes of adolescent pregnancy are early sexual debut [8, 11], lack of sexual education, lack of resources for adolescents to receive information on contraception [11], inadequate knowledge regarding the use of effective contraceptive methods, and inappropriate use of contraceptives [8]. It is estimated that 35% of the Guatemalan population are married before the age of 18 years, and 18% of girls between the ages of 15 and 19 are married or in stable unions. According to the United Nations Children's Fund (UNICEF), a lack of access to education is the main cause of these phenomena [8].

Although some previous studies have examined maternal and perinatal outcomes among adolescents, few have used a cohort study design or compared groups based on maternal age and ethnicity to identify associations that are unique to Indigenous adolescents. Our study aims to assess factors associated with pregnancy, childbirth, and perinatal outcomes among women who gave birth in a public hospital in Guatemala and to compare Indigenous adolescents with other pregnant women to understand what factors make this group more vulnerable.

## Methods

We conducted a retrospective cohort study in which we analyzed the database from the public San Juan De Dios Hospital in Guatemala. The hospital is located on the outskirts of the city of Quetzaltenango, a city with more than half a million people, most belonging to a Mayan ethnic group. The hospital provides care to people from the urban and rural areas of all western states of Guatemala; these states have similar poverty rates, Indigenous populations, and language characteristics.

The sample comprised women who gave birth at the hospital between January 2018 and June 2019. Data were collected from medical records. Moreover, the medical records also included an “antenatal control form”, which is a questionnaire adopted by the Guatemalan Ministry of Health that collects information about whether the pregnancy was planned or if it was a consequence of contraceptive failure. All reports—from antenatal care, birth, and postpartum care—were integrated.

The Ethical Committee of the Hospital San Juan de Dios, Quetzaltenango, Guatemala, and the Women’s Hospital from the State University of Campinas, Brazil authorized the study, granted permission to conduct the study, and waived the need for informed consent. The data were nonidentifiable following collection.

### Data analyses

The women were classified by age and ethnicity, forming four groups: Indigenous adolescents, Nonindigenous adolescents, Indigenous adult women, and Nonindigenous adult women.

Age was categorized as ≤ 19 years or ≥ 20 years. Ethnicity was self-reported by the women in the database. In Guatemala, there are 25 ethnic groups, 22 of which are *Maya*. Women who self-reported as belonging to the *Mayan* ethnic group were considered “Indigenous”.

We compared sociodemographic variables, gynecological and obstetric history, childbirth care data, and perinatal outcomes between indigenous adolescents and other women (Nonindigenous adolescents, Indigenous adults, and Nonindigenous adults) in a bivariate analysis using χ^2^ and Fisher’s exact test.

We performed multiple logistic regression to examine the relationship between being an Indigenous adolescent and their outcomes in comparison with other ethnic and age groups. We adjusted for variables such as education (none, primary, secondary, or university), marital status (with partner/married or single), previous pregnancy (0, 1–2, or ≥ 3), planned pregnancy (yes or no), and use of a previous contraceptive method (yes or no). We considered five perinatal risk outcomes (eclampsia, preeclampsia, 3rd trimester and postpartum hemorrhage, gestational age at birth < 37 weeks, and low birth weight) and six quality of care outcomes (mode of birth, labor augmentation, episiotomy, postpartum oxytocin use, analgesia, and local anesthesia). Crude and adjusted odds ratios with the respective 95% confidence intervals were included. The statistical software package Analytics Software & Solutions version 9.4 (SAS) for Windows was used for all statistical analyses.

## Results

Data from 8048 women who gave birth between January 2018 and June 2019 were analyzed. The sample consisted of 819 (10.2%) Indigenous adolescents, 813 (10.1%) Nonindigenous adolescents, 3324 (41.3%) Indigenous adult women, and 3092 (38.4%) Nonindigenous adult women.

Sociodemographic data and gynecological/obstetric characteristics are described in Table 1. Indigenous adolescents had fewer years of schooling than Nonindigenous adolescents (*p* < 0.001). Lack of previous contraception was more frequent in Indigenous adolescents than in Indigenous adult women (*p* = 0.007) and Nonindigenous adult women (*p* = 0.013). Single status was higher among Indigenous adolescents than among Indigenous adults (*p* < 0.001) and Nonindigenous adults (*p* < 0.001). There was no significant difference between the groups of adolescents regarding previous contraception and marital status.

**Table 1:** Comparison of sociodemographic characteristics and gynecological-obstetric history among indigenous adolescents and nonindigenous adolescents, indigenous adult women, and nonindigenous adult women

Variables	Adolescents (10–19y)			Adult Women (> 19y)				Total
	Indigenous (1)	Nonindigenous (2)	(1) vs. (2)	Indigenous (3)	(1) vs. (3)	Nonindigenous (4)	(1) vs. (4)	
	n (%)	n (%)	P-value	n (%)	P-value	n (%)	P-value	
	819 (10.18)	813 (10.10)		3324 (41.30)		3092 (38.42)		8048
Mean (standard deviation) of age	17.52 (1.40)	17.48 (1.40)		27.15 (5.61)		26.70 (5.35)		
School level^a^			< 0.001		< 0.001		< 0.001	8048
None	30 (3.66)	32 (3.94)		269 (8.09)		169 (5.47)		
Elementary (0–7y)	473 (57.75)	362 (44.63)		1882 (56.62)		1318 (42.63)		
High school (7–12y)	310 (37.85)	399 (49.08)		1060 (31.89)		1432 (46.31)		
University (> 12y)	6 (0.73)	20 (2.46)		113 (3.40)		173(5.60)		
Marriage status^a^			0.148		< 0.001		< 0.001	8048
With partner or married	692 (84.49)	676 (83.15)		3084 (92.78)		2760 (89.26)		
Without partner	125(15.26)	129 (15.87)		231 (6.95)		316 (10.22)		
Other	2 (0.24)	8 (0.98)		9 (0.27)		16 (0.52)		
Previous diabetes^b^			1.000		1.000		1.000	8040
No	818 (99.88)	812 (100)		3317 (99.82)		3084 (99.84)		
Yes	1 (0.12)	0 (0)		6 (0.18)		5 (0.16)		
Missing	3	1		1		3		8
Previous hypertension^b^			1.000		1.000		0.742	8043
No	816 (99.63)	809 (99.75)		3310(99.61)		3080 (99.68)		
Yes	3 (0.37)	2 (0.25)		13 (0.39)		10 (0.32)		
Missing	0	2		1		2		5
Previous preeclampsia^b^			0.686		0.290		0.083	8043
No	817 (99.76)	809 (99.63)		3301 (99.37)		3065 (99.19)		
Yes	2 (0.24)	3 (0.37)		21 (0.63)		25 (0.81)		
Missing	0	1		2		2		5
Previous eclampsia^b^			–		0.590		1.000	8044
No	819 (100)	812 (100)		3318 (99.85)		3 087 (99.90)		
Yes	(0)	(0)		5 (0.15)		3 (0.10)		
Missing	0	1		1		2		4
Previous cardiopathy^b^			0.248		1.000		0.217	8040
No	818 (100)	810 (99.75)		3319 (99.88)		3079 (99.74)		
Yes	(0)	2 (0.25)		4 (0.12)		8 (0.26)		
Missing	1	1		1		5		8
Previous pregnancies^a^			0.440		< 0.001		< 0.001	8048
0	638 (77.90)	654 (80.44)		1000 (30.08)		933 (30.71)		
1–3	176 (21.49)	154 (18.94)		2033 (61.16)		1930 (62.42)		
> 4	5 (0.61)	5 (0.62)		291 (8.75)		229 (7.41)		
Previous births^a^			0.608		< 0.001		< 0.001	8018
0	657 (80.71)	664 (82.08)		1064 (32.15)		1018 (33.00)		
1–2	149 (18.30)	140 (17.31)		1731(52.30)		1623 (52.61)		
≥ 3	8 (0.98)	5 (0.62)		515 (15.56)		444 (14.39)		
Missing	5	4		14		7		30
Previous c-section^a^			0.947		< 0.001		< 0.001	8048
0	753 (94.01)	746 (93.72)		2554 (78.03)		2352 (77.78)		
1	45 (5.62)	47 (5.90)		555 (16.96)		521 (17.23)		
≥ 2	3 (0.37)	3 (0.38)		164 (5.01)		151 (4.99)		
Missing	18	17		51		68		154
Planned pregnancy^a^			0.038		0.135		< 0.001	8048
No	481 (58.73)	436 (53.63)		1856 (55.84)		1465(47.38)		
Yes	338 (41.27)	377 (46.37)		1468 (44.16)		1627 (52.62)		
Previous contraception^a^			0.420		0.007		0.013	8048
No	779 (95.12)	780 (95.94)		3072 (92.42)		2865 (92.66)		
Yes	40 (4.88)	33 (4.06)		252 (7.58)		227 (7.34)		

The reference group is always indigenous adolescents (Group 1)

y: years

Analyzed with: ^a^Chi square test, ^b^Fisher’s Exact test

The antenatal care data from the four groups are presented in Table 2. More than a quarter of women did not receive antenatal care in any group (28.72% Nonindigenous adolescents, 38.30% Indigenous adults, and 26.78% Nonindigenous adults). Among Indigenous adolescents, 213 (34.6%) did not receive antenatal care. There was no significant difference between groups.

**Table 2:** Comparison of antenatal care data between indigenous adolescents and nonindigenous adolescents, indigenous adult women, and nonindigenous adult women

Antenatal care data	Adolescents (10–19y)			Adult women (> 19y)				Total
	Indigenous (1)	Nonindigenous (2)	(1) vs. (2)	Indigenous (3)	(1) vs. (3)	Nonindigenous (4)	(1) vs. (4)	
	n (%)	n (%)	p-value	n (%)	p-value	n (%)	p-value	
	819 (10.18)	813 (10.10)		3324 (41.30)		3092 (38.42)		8048
Iron supplementation^a^			0.784		0.288		0.758	7503
No	79 (10.53)	85 (10.97)		284 (9.26)		320 (10.93)		
Yes	671 (89.47)	690 (89.03)		2782 (90.74)		2609 (89.07)		
Missing	69	55		258		163		545
Folate supplementation^a^			0.174		0.175		0.310	7336
No	103 (13.96)	88 (11.61)		363 (12.12)		359 (12.55)		
Yes	635 (86.04)	670 (88.39)		2633 (87.88)		2487 (87.45)		
Missing	81	55		328		248		712
Assessment of Bacteriuria (after 20w)^a^			0.079		0.234		0.083	7871
Normal	48 (5.96)	67 (8.34)		249 (7.60)		244 (8.01)		
Abnormal	13 (1.61)	7 (0.87)		62 (1.89)		34 (1.12)		
Not done	744 (92.42)	729 (90.78)		2966 (90.51)		2768 (90.87)		
Missing	14	10		47		46		177
Syphilis—non-treponemic test (after 20w)^a^			0.519		0.410		0.019	8041
Negative	629 (76.89)	636 (78.23)		2481 (74.75)		2496 (80.75)		
Positive	0 (0)	0 (0)		2 (0.06)		6 (0.19)		
Unknown	189 (23.11)	177 (21.77)		836 (25.19))		589 (19.06)		
Missing	1	0		5		1		7
Number of ANC visits^a^			0.051		0.086		< 0.001	5798
0	213 (34.58)	166 (28.72)		941 (38.30)		575 (26.78)		
1–3	234 (37.99)	236 (40.83)		873 (35.53)		824 (38.38)		
4–7	133 (21.59)	151 (26.12)		545 (22.18)		636 (29.62)		
≥ 8	36 (5.84)	25 (4.33)		98 (3.99)		112 (5.22)		
Missing	203	235		867		945		2250
Preeclampsia^a^			0.784		0.364		0.147	8048
No	812 (99.15)	805 (99.02)		3283 (98.77)		3045 (98.48)		
Yes	7 (0.85)	8 (0.98)		41 (1.23)		47 (1.52)		
Eclampsia^b^			1.000		0.082		0.509	8048
No	815 (99.51)	809 (99.51)		3319 (99.85)		3082 (99.68)		
Yes	4 (0.49)	4 (0.49)		5 (0.15)		10 (0.32)		
1st trimester hemorrhage^b^			1.000		0.463		0.407	8044
No	816 (99.63)	810 (99.75)		3315 (99.76)		3084 (99.81)		
Yes	3 (0.37)	2 (0.25)		8 (0.24)		6 (0.19)		
Missing	0	1		1		2		4
2nd trimester hemorrhage^b^			1.000		0.357		0.506	8041
No	818 (99.88)	813 (100)		3318 (99.97)		3088 (99.94)		
Yes	1 (0.12)	0 (0)		1 (0.03)		2 (0.06)		
Missing	0	0		5		2		7
Multiple pregnancy^a^			0.801		0.704		0.536	7974
No	793 (98.88)	797 (99.01)		3230 (98.75)		3013 (99.01)		
Yes	9 (1.12)	8 (0.99)		106 (3.19)		30 (0.99)		
Missing	17	8		0		49		74

The reference group is always indigenous adolescents (Group 1)

*ANC* antenatal care, *w* weeks

Analyzed with: ^a^Chi square test, ^b^Fisher’s Exact test

Obstetric practices during childbirth are described in Table 3. We found high rates of episiotomy among Indigenous and Nonindigenous adolescents (42.5% and 38.8%, respectively) and a lower rate of cesarean delivery among Indigenous adolescents (42.6%) than among Indigenous adult women (47.9%; *p* = 0.003) and Nonindigenous adult women (47.0%; *p* = 0.023). The use of oxytocin for labor augmentation was high (> 50%) in all groups, and the incidence of incomplete expulsion of the placenta was higher among Indigenous adolescents than among Nonindigenous adults (*p* = 0.024). Nonindigenous adult women received labor analgesia more often (*p* = 0.016).

**Table 3:** Comparison of birth data between indigenous adolescents and nonindigenous adolescents, indigenous adult women, and nonindigenous adult women

Birth data	Adolescents (10–19)			Adults (> 19)				Total
	Indigenous (1)	Nonindigenous (2)	(1) vs. (2)	Indigenous (3)	(1) vs. (3)	Nonindigenous (4)	(1) vs. (4)	
	n (%)	n (%)	p-value	n (%)	p-Value	n (%)	p-value	
	819 (10.18)	813 (10.10)		3324 (41.30)		3092 (38.42)		8048
Mode of birth^b^			0.476		0.003		0.023	8048
Vaginal	470 (57.38)	454 (55.84)		1729 (52.02)		1636 (52.91)		
Cesarean	349 (42.61)	359 (44.16)		1595 (47.98)		1456 (47.09)		
Labor augmentation^a^			0.112		0.421		0.013	8032
No	382 (46.76)	347 (42.84)		1604 (48.03)		1293 (41.90)		
Yes	435 (53.24)	463 (57.16)		1715 (51.67)		1793 (58.10)		
Missing	2	3		5		6		16
3th trimester/postpartum hemorrhage^a^			0.555		0.704		0.536	8048
No	795 (97.07)	785 (96.56)		3218 (96.81)		2988 (96.64)		
Yes	24 (2.93)	28 (3.44)		106 (3.19)		104 (3.36)		
Complete placenta^a^			0.257		0.157		0.024	8018
No	28 (3.43)	20 (2.48)		84 (2.53)		64 (2.08)		
Yes	788 (96.57)	787 (97.52)		3233 (97.47)		3014 (97.92)		
Missing	3	6		7		14		30
Postpartum oxytocin^a^			0.108		0.800		< 0.001	8048
No	280 (34.19)	309 (38.01)		1152 (34.66)		1280 (41.40)		
Yes	539 (65.81)	504 (61.99)		2172 (65.34)		1812 (56.60)		
Use of antibiotics^a^			0.885		0.316		0.379	8046
No	608 (74.24)	601 (73.92)		2522 (75.92)		2248 (72.70)		
Yes	211 (25.76)	212 (26.08)		800 (24.08)		844 (27.30)		
Missing	0	0		2		0		2
Vaginal birth position^a^			0.191		0.715		0.734	4286
Sitting	28 (5.96)	16 (3.53)		92 (5.32)		84 (5.14)		
Squatting	5 (1.06)	4 (0.88)		25 (1.45)		21 (1.28)		
Lying	437 (92.98)	433 (95.58)		1611 (93.23)		1530 (93.58)		
Missing	0	1		1		1		3
Episiotomy (vaginal birth)^a^			0.251		< 0.001		< 0.001	
No	269 (57.48)	276 (61.20)		1407 (81.42)		1347 (82.49)		4280
Yes	199 (42.52)	175 (38.80)		321 (18.58)		286 (17.51)		
Missing	2	3		1		3		9
Vaginal tears (vaginal birth)^b^			0.387		0.518		0.350	4160
No	442 (98.00)	428 (97.05)		1634 (97.44)		1542 (96.92)		
Grade 1	6 (1.33)	8 (1.81)		32 (1.91)		33 (2.07)		
Grade 2	2 (0.44)	5 (1.13)		10 (0.60)		15 (0.94)		
Grade 3	1 (0.22)	0 (0)		1 (0.06)		1 (0.06)		
Missing	19	13		52		45		129
Analgesia^a^			0.256		0.377		0.016	8048
No	714 (87.18)	693 (85.24)		2935 (88.30)		2590 (83.76)		
Yes	105 (12.82)	120 (14.76)		389 (11.70)		502 (16.24)		
Local anesthesia^a^			0.378		< 0.001		< 0.001	8048
No	702 (85.71)	709 (87.21)		3131 (94.19)		2863 (92.59)		
Yes	117 (14.29)	104 (12.79)		193 (5.81)		229 (7.41)		
General anesthesia^a^			0.820		0.735		0.905	8031
No	805 (98.65)	798 (98.52)		3266 (98.49)		3049 (98.71)		
Yes	11 (1.35)	12 (1.48)		50 (1.51)		40 (1.29)		
Missing	3	3		8		3		17
Blood transfusion^b^			1.000		0.804		0.779	8041
No	813 (99.51)	805 (99.51)		3303 (99.37)		3077 (99.55)		
Yes	4 (0.49)	4 (0.49)		21 (0.63)		14 (0.45)		
Missing	2	4		0		1		7
Attended the birth^a^			0.212		0.372		0.978	8010
Physician	693 (84.72)	702 (86.88)		2842 (85.94)		2608 (84.76)		
Other professional	125 (15.28)	106 (13.12)		465 (14.06)		469 (15.24)		
Missing	1	5		17		15		38

The reference group is always indigenous adolescents (Group 1)

Analyzed with: ^a^Chi square test, ^b^Fisher’s exact test

Neonatal results are described in Table 4. The frequency of low birth weight was higher among Nonindigenous adolescents than among Indigenous adolescents (*p* = 0.009). Low 5-min Apgar scores were more frequent among Nonindigenous adolescents (*p* = 0.027), Nonindigenous adults (*p* = 0.007), and Indigenous adults (*p* = 0.009) compared with Indigenous adolescents. Neonatal resuscitation with intubation was more frequent among Nonindigenous adults (*p* = 0.008) and Indigenous adults (*p* = 0.021) than among Indigenous adolescents. We did not observe differences in the prematurity rate between groups.

**Table 4:** Comparison of neonatal outcomes among indigenous adolescents and nonindigenous adolescents, indigenous adult women, and nonindigenous adult women

Neonate data	Adolescents (10–19y)			Adult women (> 19y)				Total
	Indigenous (1)	Nonindigenous (2)	(1) vs. (2)	Indigenous (3)	(1) vs. (3)	Nonindigenous (4)	(1) vs. (4)	
	n (%)	n (%)	p-value	n (%)	p-value	n (%)	p-value	
	819 (10.18)	813 (10.10)		3324 (41.30)		3092 (38.42)		8048
Status at birth^b^			0.123		0.161		0.133	8047
Alive	817 (99.76)	808 (99.38)		3299 (99.28)		3074 (99.42)		
Any fetal death	1 (0.12)	5 (0.51)		21 (0.63)		16 (0.52)		
Death Ignored^c^	1 (0.12)	0 (0)		3 (0.09)		2 (0.06)		
Missing	0	0		1		0		1
Birth weight (g)^a^			0.009		0.035		0.145	8048
< 2500	114 (13.92)	152 (18.70)		463 (13.93)		456 (14.75)		
2500–3999	705 (86.08)	661 (81.30)		2834 (85.26)		2623 (84.23)		
≥ 4000	0 (0)	0 (0)		27 (0.81)		13 (0.42)		
Gestational age (weeks)^a^			0.156		0.976		0.212	8048
< 37	67 (8.18)	83 (10.21)		273 (8.21)		297 (9.61)		
≥ 37	752 (91.82)	730 (89.79)		3051 (91.79)		2795 (90.39)		
Apgar 5th min^a^			0.027		0.009		0.007	8043
< 7	4 (0.49)	13 (1.60)		57 (1.72)		55 (1.78)		
≥ 7	814 (99.51)	799 (98.40)		3265 (98.28)		2036 (98.22)		
Missing	1	1		2		1		5
Resuscitation/oxygen^a^			0.697		0.276		0.126	8044
No	805 (98.41)	800 (98.64)		3251 (97.80)		3014 (97.51)		
Yes	13 (1.59)	11 (1.36)		73 (2.20)		77 (2.49)		
Missing	1	2		0		1		4
Resuscitation/intubation^a^			0.177		0.021		0.008	
No	816 (99.76)	806 (99.26)		3287 (98.89)		3050 (98.67)		8045
Yes	2 (0.24)	6 (0.74)		37 (1.11)		41 (1.33)		
Missing	1	1		0		1		3
Newborn’s reception^a^			0.205		0.686		0.712	7974
Physician	804 (98.77)	797 (99.38)		3256 (98.94)		3034 (98.92)		
Other	10 (1.23)	5 (0.62)		35 (1.06)		33 (1.08)		
Missing	5	11		33		25		74
Birth defects^b^			0.623		1.000		0.477	8042
No	817 (99.88)	810 (99.75)		3316 (99.76)		3078 (99.68)		
Yes	1 (0.12)	2 (0.25)		8 (0.24)		10 (0.32)		
Missing	1	1		0		4		6
Referred^b^			0.558		0.637		0.271	7945
Mother-baby room	791 (97.53)	786 (97.64)		3197 (97.41)		2985 (97.97)		
Neonatal intensive care	18 (2.22)	19 (2.36)		69 (2.10)		60 (1.97)		
Other hospital	2 (0.25)	0 (0)		16 (0.49)		2 (0.07)		
Missing	8	8		42		45		103

The reference group is always indigenous adolescents (Group 1)

Analyzed with: ^a^Chi square test, ^b^Fisher’s Exact test; ^c^death ignored: moment of fetal or newborn death was ignored

**Fig. 1:**
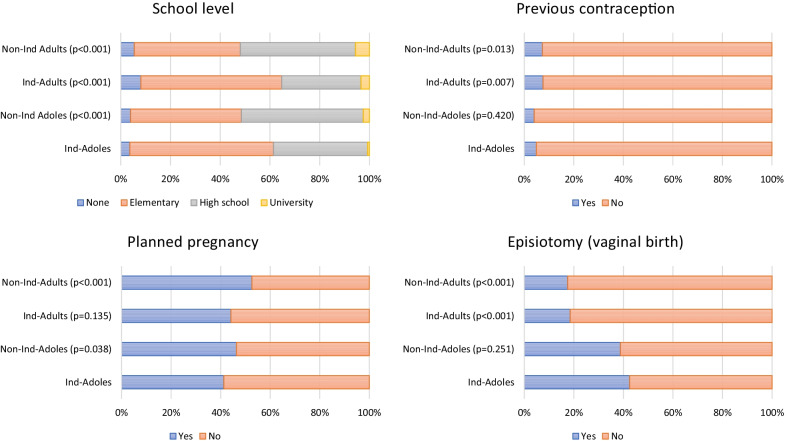
Comparison of school level, planned pregnancy, use of previous contraception, and episiotomy among Indigenous adolescents and non-indigenous adolescents, indigenous adults, and non-indigenous adults women in a Public Hospital in Guatemala

Figure 1 summarizes some of the differences between the groups.

The multivariate analysis evaluated outcomes regarding perinatal risk and quality of care among the groups based on age and ethnicity. Nonindigenous adolescents had more newborns with low birth weight than Indigenous adolescents (OR: 1.44 [95% CI 1.10–1.87]). Indigenous adults and non-Indigenous adults had fewer instances of episiotomy (OR: 0.60 [95% CI 0.49–0.74] and OR: 0.56 [95% CI 0.45–0.70], respectively]) and local anesthesia (OR: 0.59 [95% CI: 0.46–0.76] and OR: 0.77 [95% CI: 0.60–0.99], respectively) than Indigenous adolescents. Nonindigenous adults received more analgesia than Indigenous adolescents (OR: 1.36 [95% CI 1.07–1.73]). Indigenous adults and Nonindigenous adults had more instances of cesarean delivery than Indigenous adolescents (OR: 1.47 [95% CI 1.25–1.73] and OR: 1.39 [95% CI 1.18–1.64], respectively) (Table 5).

**Table 5:** Multiple logistic regression with crude and adjusted analysis of outcomes (perinatal risk and quality of care) according to group of age and ethnicity

Groups	Outcomes (perinatal risk)									
	Eclampsia		Preeclampsia		3rd trimester/postpartum hemorrhage		Gestational age at birth (< 37 week)		Low birth weight (< 2500 g)	
	OR crude (95% CI)	OR adjusted (95% CI)	OR crude (95% CI)	OR adjusted (95% CI)	OR crude (95% CI)	OR adjusted (95% CI)	OR crude (95% CI)	OR adjusted (95% CI)	OR crude (95% CI)	OR adjusted (95% CI)
Indigenous adolescent	1.00	1.00	1.00	1.00	1.00	1.00	1.00	1.00	1.00	1.00
Nonindigenous adolescent	1.01 (0.25–4.04)	0.95 (0.23–3.89)	1.15 (0.42–3.19)	1.18 (0.43–3.28)	1.18 (0.68–2.06)	1.21 (0.69–2.11)	1.28 (0.91–1.79)	1.28 (0.91–1.79)	**1.42 (1.09–1.85)**	**1.44 (1.10–1.87)**
Indigenous adult women	0.31 (0.08–1.15)	0.42 (0.11–1.68)	1.45 (0.65–3.24)	1.76 (0.77–4.04)	1.09 (0.70–1.71)	0.98 (0.61–1.58)	1.00 (0.76–1.33)	0.97 (0.73–1.30)	1.00 (0.80–1.25)	0.99 (0.79–1.25)
Nonindigenous adult women	0.66 (0.21–2.11)	1.06 (0.31–3.65)	1.79 (0.81–3.98)	2.22 (0.97–5.06)	1.15 (0.74–1.81)	1.05 (0.65–1.70)	1.19 (0.90–1.57)	1.17 (0.87–1.56)	1.07 (0.86–1.34)	1.09 (0.87–1.38)

Groups	Outcomes (quality of care)											
	Mode of birth (cesarean)		Labor augmentation		Episiotomy		Local anesthesia		Postpartum oxytocin		Analgesia	
	OR crude (95% CI)	OR adjusted (95% CI)	OR crude (95% CI)	OR adjusted (95% CI)	OR crude (95% CI)	OR adjusted (95% CI)	OR crude (95% CI)	OR adjusted (95% CI)	OR crude (95% CI)	OR adjusted (95% CI)	OR crude (95% CI)	OR adjusted (95% CI)
Indigenous adolescent	1.00	1.00	1.00	1.00	1.00	1.00	1.00	1.00	1.00	1.00	1.00	1.00
Nonindigenous adolescent	1.07 (0.88–1.30)	1.04 (0.86–1.27)	1.17 (0.96–1.42)	1.12 (0.92–1.36)	0.86 (0.68–1.08)	0.82 (0.65–1.04)	0.88 (0.66–1.17)	0.87 (0.66–1.17)	0.85 (0.69–1.04)	0.86 (0.70–1.05)	1.18 (0.89–1.56)	1.14 (0.86–1.51)
Indigenous adult women	**1.24 (1.07–1.45)**	**1.47 (1.25–1.73)**	0.94 (0.81–1.10)	0.96 (0.82–1.13)	**0.33 (0.27–0.41)**	**0.60 (0.49–0.74)**	**0.37 (0.29–0.47)**	**0.59 (0.46–0.76)**	0.98 (0.83–1.15)	0.99 (0.84–1.18)	0.90 (0.72–1.14)	0.98 (0.77–1.24)
Nonindigenous adult women	**1.20 (1.03–1.40)**	**1.39 (1.18–1.64)**	**1.22 (1.04–1.42)**	1.17 (0.99–1.38)	**0.32 (0.26–0.39)**	**0.56 (0.45–0.70)**	**0.48 (0.38–0.61)**	**0.77 (0.60–0.99)**	**0.74 (0.63–0.86)**	**0.76 (0.64–0.90)**	**1.32 (1.05–1.65)**	**1.36 (1.07–1.73)**

Statistically significant values are in bold

Model adjusted by variables: education (none, primary, secondary, university), marital status (with partner or married, without partner, other), previous pregnancy (0, 1–2, ≥ 3), planned pregnancy (yes or no) and use previous contraceptive method (yes or no)

## Discussion

Our results showed that Indigenous adolescents, compared with other Indigenous and Nonindigenous women who gave birth in a public hospital in Guatemala, had less education, were more likely to be single, had more unplanned pregnancies, had less use of previous contraception, and had fewer antenatal care visits. Beyond a few variables, we found insignificant variations in intervention coverage and neonatal outcomes between groups. Indigenous adolescents received more postpartum oxytocin than Nonindigenous adolescents. They also received more episiotomies and local anesthesia than adults overall. Other studies have shown that adolescent mothers and their children have an increased risk of adverse health outcomes [12, 13]; in our study, substantial differences regarding perinatal risk and quality of care were not observed among the groups based on age and ethnicity, except for episiotomy and local anesthesia.

Several reports from Latin America and the Caribbean regions have shown that Indigenous women are more likely to live in conditions of poverty [14, 15], have fewer years of formal education, and are more likely to live in rural areas [14, 15], which reflects the marginalization and economic and social inequity they experience. These findings are reflected in health care indicators in which Indigenous women are reported to have less access to quality care during childbirth [16–18], less contraceptive coverage, and higher maternal and infant mortality [16]. From this perspective, it is important to understand the social determinants of health and the intersection of categories such as gender, ethnicity, and age and how they place Indigenous adolescents in vulnerable situations [15].

Likewise, our results also contrast with those studies that demonstrated health disparities among the Indigenous population [19, 20]. However, it is necessary to analyze these findings carefully. Most of these studies included other relevant socioeconomic variables, such as income level [20], distance from healthcare centers [20], and sociocultural factors [19]. For instance, a study regarding the quality of care during pregnancy and childbirth among Guatemalan rural women, including markers of ethnicity, language, and dress, showed that Indigenous women who speak Spanish and wear Western clothing had more similarities in the quality of care during pregnancy and childbirth with non-Indigenous women than to Indigenous women [19].

According to our findings, Indigenous adolescents have fewer years of schooling than non-Indigenous adolescents. These data corroborate the findings of national data that showed higher levels of absenteeism among Indigenous adolescents than among their non-Indigenous counterparts [10].

The lower participation of adolescents in the education system may represent a barrier to accessing sexual education programs, and this aspect may influence the results found in our study, especially the higher prevalence of unplanned pregnancies and lower use of contraceptive methods in this population [12, 16].

Regarding unplanned pregnancies, our data suggest that this issue was higher among Indigenous adolescents. In line with our findings, national statistics about family planning needs have shown that the use of modern methods of contraception increases with age and only 8% of girls between 15 and 19 years old use these methods [21]. The use of contraceptive methods, issues associated with gender discrimination (e.g., the reproduction of the stereotype that a woman’s role is based on reproduction and family care), and the lack of women’s autonomy in decision-making regarding family planning can influence the infrequent use of these methods [18, 22, 23]. Scientific evidence shows that barriers that limit the use of contraception in adolescents persist even after they become mothers [12, 22].

Our data showed interesting results regarding single motherhood, especially among adolescents rather than adults. In Latin America, changes in family structure and living arrangements are observed with an increase in single parenthood, especially among women, and there is an association between being a single mother and income inequalities [24]. Further qualitative studies are needed to understand patterns in childbearing outside of union among adolescents and the stigmas experienced by pregnant unwed adolescents.

Furthermore, data regarding antenatal care have shown that 70.5% of adolescents started attending follow-up from the second trimester of pregnancy compared with only 26.8% in the first trimester of pregnancy [25]. Moreover, increasing the available knowledge about the causes of adolescent pregnancy would make it possible to create appropriate initiatives for each group in the vulnerable population.

Our findings on childbirth practices show higher use of episiotomy among Indigenous adolescents. Although this practice is decreasing worldwide, it is still observed at varied rates among global regions [26]. For instance, a study conducted in Mexican Indigenous communities showed that one-third of women who delivered in a healthcare facility reported having an episiotomy [27]. Factors that are associated with the use of episiotomy include primiparas women, epidural analgesia, and newborn weight > 4000 g [28]. Some clinicians believe that routine episiotomy prevents severe perineal tears, but this belief is not supported by current relevant evidence [29]. The prevalence of interventionist practices observed in our sample, such as the use of episiotomy specifically among adolescents, requires further analysis.

A similar situation was observed regarding analgesia use during childbirth. Interestingly, among Indigenous adolescents, we observed less use of analgesia during childbirth and more use of local anesthesia. Regarding this topic, various pain management strategies exist that include nonpharmacological, pharmacological, and regional analgesia [30]. However, our study does not explore the decision-making of women and their adherence to medical recommendations on the use of these methods. Nevertheless, access to qualified information about available pain relief methods, concerns, and birth experience expectations are needs that should be addressed during prenatal care [31].

In addition, Indigenous adolescents show differences regarding cesarean section when compared with adults. Our study showed high rates of cesarean section among all participants when compared with the national statistics that showed 32.5% of deliveries were cesarean section in Quetzaltenango [21]. Our study shows the need to address the high rates of cesarean section in Guatemala overall given that the World Health Organization (WHO) recommends a maximum cesarean section rate of 15% [32].

Comparing the use of oxytocin for labor augmentation among Indigenous adolescents and non-Indigenous adults, less use was observed in Indigenous adolescents. However, among the four observed groups, more than half of the cases used oxytocin for augmentation of labor.

When comparing Indigenous adolescents with Nonindigenous adults, less postpartum oxytocin use was observed among non-Indigenous adults. The use of intramuscular or intravenous postpartum oxytocin for the prevention of postpartum hemorrhage after vaginal birth is a practice recommended by the WHO under specific conditions [33]. Meanwhile, the WHO has not recommended interventionist practices such as episiotomy and labor augmentation, considering these interventions a barrier to woman-centered care [34].

Such guidelines coincide with findings from one study on Indigenous Mexican women in which preferences and perceptions about labor and birth were discussed, and they observed that some medical practices can cause discomfort, decreasing the trust between women and their healthcare providers. This corroborates the need to consider cultural aspects [35] and current guidelines during care. In our data, we observed unnecessary interventions across all groups, such as labor augmentation, but more pointedly, we observed a prevalence of non-recommended practices among Indigenous adolescents, especially episiotomy. This can lead to a rejection of health services, particularly when these practices are not culturally accepted.

Regarding perinatal risk outcomes, Nonindigenous adolescents had more newborns with low birth weight than Indigenous adolescents. However, low birth weight was not substantially different among the groups. Similarly, national data showed that 15% of newborns had less than 2500 g, and there was no observed difference between the Indigenous and Nonindigenous populations [21].

Historically, Indigenous populations have experienced social exclusion and discrimination, issues that could be reflected in the practices of professionals within health institutions [15, 16, 22]. Therefore, it is necessary to conduct studies that enable an understanding of the relationship between health care professionals and the Indigenous population, especially concerning the care that is offered to this group during childbirth. In this sense, the Guatemalan government developed regulations to improve the health and quality of life of women and their newborns. Some of these strategies included a program for the extension of health services coverage [36], implementation of maternity homes in remote places [37], training of traditional midwives [38], and the introduction of vertical childbirth in health services, a practice adopted by this population for generations [39].

Consequently, in Guatemala, it was established that every woman would be treated in her mother language to ensure that the treatment and communication procedures were comprehensible and clear for the mother and her family [40]. Specifically, in the hospital where our data were collected, most of the care is offered in Spanish because the hospital provides care to different cities in the west of the country; having medical personnel who have mastered the native language of the residents is almost impossible. This situation has been slowly changing over time.

The main strength of our study is that it has a cohort design covering the registration data of a large population of Indigenous and Nonindigenous adolescents and women in a public hospital. Nevertheless, this approach has some limitations. Analyses were conducted using data from an existing database, which raises questions related to the completeness of the information and the generation of missing values. In addition, the exclusion of women who did not have a hospital birth could limit the observations of the sociodemographic, maternal, and perinatal characteristics of women who face greater difficulties in accessing health services. Considering the ethnic and cultural diversity that characterizes the Indigenous population, it is necessary to extend the studies to other regions. Furthermore, the quantitative approach excluded the possibility of listening to the women and collecting their subjective perceptions of the whole process, from the confirmation of pregnancy to care during childbirth. This perspective could contribute to the identification of the women’s profiles; however, this element may be useful in the development of strategies and programs aimed at this specific population.

Our data also highlight the importance of analyzing the sociodemographic characteristics and maternal and perinatal outcomes of Indigenous women treated in public health facilities to develop strategies to improve satisfaction among patients, increase the rate of return, and provide women-centered care in these health facilities. However, our data were limited to users of public health facilities. In this regard, we considered it important to explore the sexual and reproductive health needs of Indigenous adolescents who did not have access to health care services. The analysis of a single region and only women who had access to public health facilities constitutes a limitation for the generalization of our results.

Another essential consideration is our analysis of the adolescent groups. We considered adolescents to be all individuals aged 10–19 years, using the categorization recommended by the WHO [13]. However, the authors recognize that this category is a social construct and that it acquires a multiplicity of meanings for each social context, mainly concerning young Indigenous mothers. Nevertheless, this quantitative approach is limited to a comprehensive understanding of the sociocultural context in which our data were collected.

Our findings are particularly important to the development of future research about family planning and maternal outcomes among Indigenous adolescents in Guatemala and other countries in similar situations. There are several important outcomes related to family planning coverage that must be observed by health care providers considering that rapid repeat pregnancy is frequent among younger mothers [34]. Future research on contraceptive method preferences, satisfaction with antenatal care services, and analgesia decision-making in this population will be useful to enhance women-centered care approaches.

## Conclusion

In conclusion, our study revealed that Indigenous adolescents who gave birth at the San Juan De Dios Hospital had some social vulnerabilities; however, when compared to other women, similarities are observed regarding some childbirth practices and maternal and perinatal outcomes. It is essential to take into consideration the characteristics of the Indigenous population in Guatemala for the design and implementation of public policies that respect women’s sexual and reproductive rights, practices, and cultural identities. It is necessary to identify the health issues that affect adolescents and Indigenous women and ensure their active participation in public agendas.

The results of this research show that Indigenous adolescents who gave birth in a public hospital were more likely to be single at the time of pregnancy and to have attended fewer years of school than Nonindigenous adolescents. Unplanned pregnancies were more common among Indigenous adolescents, and some of them underwent obstetric practices during delivery that are not recommended, such as episiotomy.

This research provides a broad overview of the situation of pregnant women in western Guatemala. Based on these results, activities and policies can be developed that ensure equal opportunities for different ethnic groups in a way that makes these resources understandable, acceptable, and usable for this population.

## Data Availability

Data are available on request from the corresponding author.

## Abbreviations

SAS: Analytics Software & Solutions
OR: Odds ratio
OSAR: The Reproductive Health Observatories Network
UNICEF: United Nations Children’s Fund
WHO: World Health Organization
